# Different Human Copper-Zinc Superoxide Dismutase Mutants, SOD1^G93A^ and SOD1^H46R^, Exert Distinct Harmful Effects on Gross Phenotype in Mice

**DOI:** 10.1371/journal.pone.0033409

**Published:** 2012-03-16

**Authors:** Lei Pan, Yasuhiro Yoshii, Asako Otomo, Haruko Ogawa, Yasuo Iwasaki, Hui-Fang Shang, Shinji Hadano

**Affiliations:** 1 Department of Molecular Life Sciences, Tokai University School of Medicine, Isehara, Kanagawa, Japan; 2 The Institute of Medical Sciences, Tokai University, Isehara, Kanagawa, Japan; 3 Department of Neurology, Toho University Omori Medical Center, Ota-ku, Tokyo, Japan; 4 Research and Development Division, Teaching and Research Support Center, Tokai University, Isehara, Kanagawa, Japan; 5 Department of Neurology, West China Hospital, Sichuan University, Chengdu, Sichuan, China; 6 Research Center for Brain and Nervous Diseases, Tokai University Graduate School of Medicine, Isehara, Kanagawa, Japan; Cedars-Sinai Medical Center, Maxine-Dunitz Neurosurgical Institute, United States of America

## Abstract

Amyotrophic lateral sclerosis (ALS) is a heterogeneous group of fatal neurodegenerative diseases characterized by a selective loss of motor neurons in the brain and spinal cord. Creation of transgenic mice expressing mutant Cu/Zn superoxide dismutase (SOD1), as ALS models, has made an enormous impact on progress of the ALS studies. Recently, it has been recognized that genetic background and gender affect many physiological and pathological phenotypes. However, no systematic studies focusing on such effects using ALS models other than *SOD1^G93A^* mice have been conducted. To clarify the effects of genetic background and gender on gross phenotypes among different ALS models, we here conducted a comparative analysis of growth curves and lifespans using congenic lines of *SOD1^G93A^* and *SOD1^H46R^* mice on two different genetic backgrounds; C57BL/6N (B6) and FVB/N (FVB). Copy number of the transgene and their expression between *SOD1^G93A^* and *SOD1^H46R^* lines were comparable. B6 congenic mutant SOD1 transgenic lines irrespective of their mutation and gender differences lived longer than corresponding FVB lines. Notably, the G93A mutation caused severer disease phenotypes than did the H46R mutation, where *SOD1^G93A^* mice, particularly on a FVB background, showed more extensive body weight loss and earlier death. Gender effect on survival also solely emerged in FVB congenic *SOD1^G93A^* mice. Conversely, consistent with our previous study using B6 lines, lack of *Als2*, a murine homolog for the recessive juvenile ALS causative gene, in FVB congenic *SOD1^H46R^*, but not *SOD1^G93A^*, mice resulted in an earlier death, implying a genetic background-independent but mutation-dependent phenotypic modification. These results indicate that SOD1^G93A^- and SOD1^H46R^-mediated toxicity and their associated pathogenic pathways are not identical. Further, distinctive injurious effects resulted from different SOD1 mutations, which are associated with genetic background and/or gender, suggests the presence of several genetic modifiers of disease expression in the mouse genome.

## Introduction

Amyotrophic lateral sclerosis (ALS) is an inexorable neuromuscular disorder characterized by progressive loss of motor neurons in the spinal cord, brainstem and motor cortex [1]. Most ALS patients become severely paralyzed and die within 3–5 years after diagnosis. The majority of patients are sporadic (sALS), while 5–10% are familial cases (fALS), among which approximately 15–20% are associated with mutations in the gene encoding Cu/Zn superoxide dismutase (SOD1) [2]. Patients with sALS and mutant SOD1-linked fALS share many clinical and pathological features [3], [4]. Importantly, recent studies have highlighted that not only mutant SOD1 in fALS but also wild-type SOD1 can be pathogenic in sALS patients [5], [6], illuminating a possible SOD1-dependent pathogenic mechanism common to sALS and fALS.

To date, more than 160 mutations scattered throughout the SOD1 protein have been identified in fALS (http://alsod.iop.kcl.ac.uk/als/). Although mutant SOD1-mediated neuronal toxicity appears to account for disease expression [7], the exact mechanism by which mutant SOD1 impairs neuronal function leading to motor neuron death remains unclear, let alone the clinical heterogeneity; e.g. age at onset and disease duration, seen within and/or among *SOD1*-linked families [3], [8], [9]. Thus far, it is generally thought that in addition to the toxic entities associated with different *SOD1* mutations, a complex interplay between other genetic and environmental factors results in symptomatic variability in *SOD1*-linked fALS.

Genetically engineered mice have played a pivotal role not only in the molecular pathogenesis but also in the development of therapeutics for many genetic as well as non-genetic diseases. Indeed, the creation of ALS animal models, namely transgenic mice expressing a mutant SOD1, has made an enormous impact on progress of the ALS studies [4], [10], [11]. More than 10 different lines of transgenic mouse carrying a different SOD1 mutation, all of which show a selective degeneration of spinal motor neurons, have been established [4], [11]. Interestingly, the onset and progression of disease phenotypes appear to vary from line to line [12], [13]. Among these lines, animals carrying a G93A SOD1 mutation (*SOD1^G93A^*) [10] were most widely used and extensively characterized. Although a copy number of the *SOD1^G93A^* transgene, and thus expression level of the SOD1^G93A^ protein, is a major determinant of disease severity [14], genetic background and gender may also affect the *SOD1^G93A^*-linked symptoms in mice [15]–[17]. It is conceivable that there are some common genetic modifiers affecting to disease expression in mutant SOD1-expressing ALS mouse models [15], [16]. However, with the use of only a limited number of lines; i.e. *SOD1^G93A^*, such notions still remain inconclusive. Further, although it is generally thought that the different mutant SOD1 proteins are likely to cause motor neuron disease by a similar mechanism, this idea has also yet to be fully proven.

Recently, we have generated two congenic mouse lines carrying either *SOD1^G93A^* or a H46R mutation (*SOD1^H46R^*) on a C57BL/6N (B6) background [18], and crossed those mice to B6 congenic mice lacking *Als2*, a murine homolog for the causative gene for a number of recessive juvenile motor neuron diseases (MNDs) [19], generating B6 congenic ALS2-deficient *SOD1^G93A^* and *SOD1^H46R^* mice [18]. The *SOD1^H46R^* mutation accounts for a mild form of familial ALS that was originally identified in Japanese kindred [20], [21] and characterized by unusually extended disease duration after onset [3], [21]. Surprisingly, lack of *Als2* in B6 congenic *SOD1^H46R^*, but not *SOD1^G93A^*, mice results in an earlier death [18]. In addition, loss of *Gfap*, a gene encoding glial fibrillary acidic protein (GFAP), in B6 congenic *SOD1^H46R^*, but not *SOD1^G93A^*, mice also accelerates the disease progression [22]. Thus, it is possible that the *SOD1^G93A^* and *SOD1^H46R^*-mediated pathogenic mechanisms are not the same. However, at this stage, we could not formally exclude the possibility that these phenomena are unique to mice on a B6 background.

In this study, to further clarify the phenotype variability among different mutant SOD1-expressing ALS mouse models on different genetic backgrounds, we newly generated congenic lines of *SOD1^G93A^* and *SOD1^H46R^* mice as well as those lacking *Als2* on a FVB/N (FVB) background, and conducted a comparative analysis of gross phenotypes in these mutants with different genetic backgrounds.

## Results

### Copy numbers of the transgene in *SOD1^G93A^* and *SOD1^H46R^* transgenic mouse lines with different genetic backgrounds

**Figure 1:**
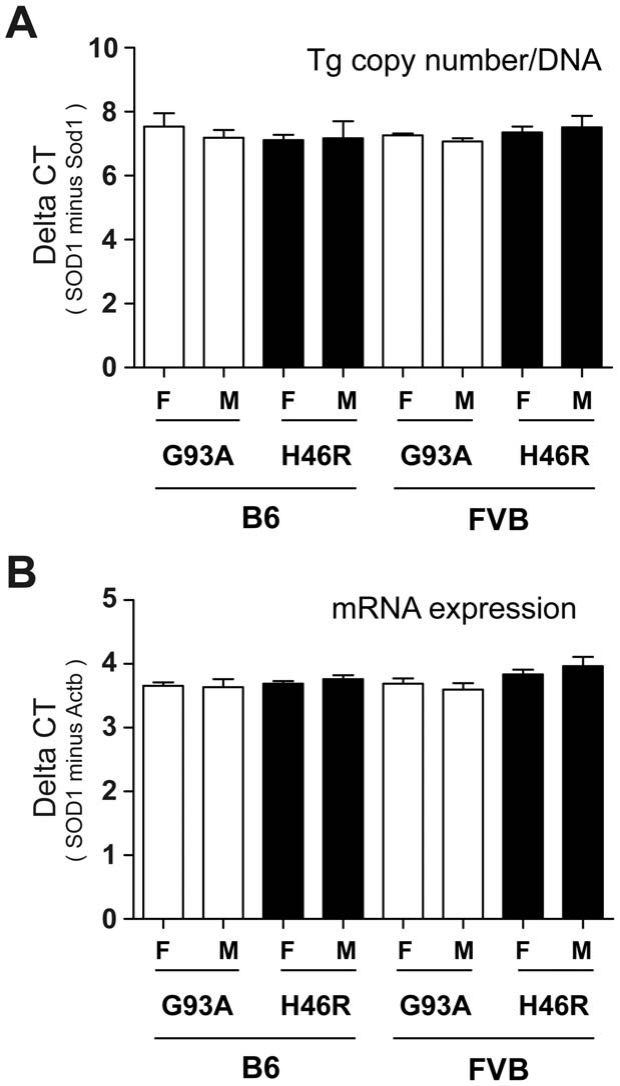
Copy numbers of the transgene and the levels of its transcript. (A) Comparison of the difference in threshold cycle (ΔCT) between the human *SOD1* transgene (SOD1) and a reference mouse *Sod1* gene (Sod1) in *SOD1^G93A^* and *SOD1^H46R^* transgenic mice. There are no significant differences in the mean values between groups with different genders (F; female, M; male), genotypes (G93A; *SOD1^G93A^*, H46R; *SOD1^H46R^*), and genetic backgrounds (B6; C57BL/6, FVB; FVB/N). (B) Comparison of the ΔCT between the human *SOD1* and the mouse *Actb* transcripts in *SOD1^G93A^* and *SOD1^H46R^* transgenic mice. There are no significant differences in the mean values between groups with different genders, genotypes, and genetic backgrounds. All values are mean ± SD (n = 4). Statistical significance is evaluated by ANOVA with Bonferroni's *post hoc* test.

In this study, we generated four independent congenic mouse lines expressing the human mutated *SOD1* gene; i.e., C57BL/6N congenic *SOD1^G93A^* (B6_G93A) and *SOD1^H46R^* (B6_H46R), and FVB/N congenic *SOD1^G93A^* (FVB_ G93A) and *SOD1^H46R^* (FVB_ H46R). The transgene in each mouse line was transmitted in the expected Mendelian ratio of an autosomal gene (data not shown). The previous studies have demonstrated that the estimated copy numbers of *SOD1^G93A^* and *SOD1^H46R^* in original transgenic lines are approximately 24 [14] and 20 [23], respectively. Since it has been shown that a copy number of the mutated *SOD1* transgene affects the disease severity [14], we first analyzed the copy numbers of the transgene in our mouse lines by quantitative PCR. The relative number of transgene's copy was estimated by the difference in threshold cycle (ΔCT, delta CT) between the transgene (*SOD1^G93A^* or *SOD1^H46R^*) and control (mouse *Sod1*). There were no significant differences in the ΔCT values among all four lines with different transgenes, genetic backgrounds, and/or genders (Figure 1A and Table 1). These results indicate that each transgene locus retains comparable number of the mutated *SOD1* gene that is stably transmitted in the course of generating our congenic lines, and that the copy numbers of the transgene between *SOD1^G93A^* and *SOD1^H46R^* are almost equal.

**Table 1:** Summary of the quantitative analysis of the transgenes and their expression.

Strain	SOD1mutant	Gender	n	Tg copy number	mRNA	Protein
				(ΔCt)*a	(ΔCt)*b	SOD1/β-actin ratio
B6	G93A	F	4	7.50±0.80	3.65±0.11	1.04±0.12
		M	4	7.20±0.50	3.64±0.25	0.99±0.29
B6	H46R	F	4	7.10±0.30	3.69±0.08	0.98±0.13
		M	4	7.20±1.10	3.76±0.13	1.15±0.48
FVB	G93A	F	4	7.30±0.10	3.69±0.17	0.95±0.30
		M	4	7.10±0.21	3.60±0.20	0.92±0.05
FVB	H46R	F	4	7.40±0.34	3.83±0.16	1.09±0.08
		M	4	7.50±0.70	3.96±0.29	1.01±0.24

Values are mean ± SD.

*a ΔCt = (Ct for human SOD1)−(Ct for mouse Sod1).

*b ΔCt = (Ct for human SOD1 mRNA)−(Ct for β-actin mRNA).

### Levels of the transgene transcripts in *SOD1^G93A^* and *SOD1^H46R^* transgenic mouse lines with different genetic backgrounds

To investigate whether the differences in mutations, genetic backgrounds, and/or genders affect the expression levels of the mutated *SOD1* transcript, we performed a quantitative reverse transcriptase (qRT)-PCR using total RNA from the spinal cord of mice at a pre-clinical stage (12 weeks of age). Although the levels of transcript for *SOD1^H46R^* relative to the β-actin mRNA (*Actb*) showed a higher tendency when compared to those for *SOD1^G93A^*, there were no significant differences in the levels of mutated *SOD1* mRNA among different transgenic lines used in this study (Figure 1B and Table 1). These data indicate that expression levels of the mutated *SOD1* transcripts are affected neither by difference in mutations, genetic backgrounds, nor genders in mice.

### Levels of the mutant SOD1 protein in *SOD1^G93A^* and *SOD1^H46R^* transgenic mouse lines with different genetic backgrounds

**Figure 2:**
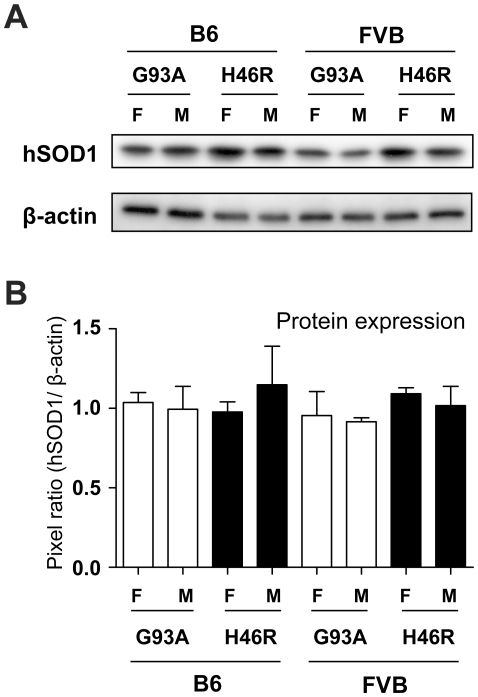
Comparisons of the mutant SOD1 protein levels in different mutant *SOD1* transgenic mouse lines. (A) Representative data for western blot analysis of the human mutant SOD1 proteins (hSOD1). The 1% Triton X100-soluble fractions of the spinal cord from mice at 12 weeks of age with different genders (F; female, M; male), genotypes (G93A; *SOD1^G93A^*, H46R; *SOD1^H46R^*), and genetic backgrounds (B6; C57BL/6, FVB; FVB/N) are analyzed. β-actin serves as control. (B) Quantitative analyses of western blotting for mutant SOD1 in the spinal cord from 12 week-old mice. Densitometric data for immunoreactive signals are normalized by the levels of β-actin. There are no significant differences in the mean values between groups with different genders, genotypes, and genetic backgrounds. Values are mean ± SEM (n = 4) in an arbitrary unit. Statistical significance is evaluated by ANOVA with Bonferroni's *post hoc* test.

To examine whether differences in mutations, genetic backgrounds, and/or genders affect the expression levels of the mutant SOD1 protein, we next performed western blot analysis of the spinal cord extracts obtained from mice at a pre-clinical stage (12 weeks of age) using anti-human SOD1 antibody. Although the levels of the mutant SOD1 proteins slightly varied (Figure 2A and 2B), a quantitative analysis revealed no statistical differences in the mean values among all tested mouse lines (Figure 2B and Table 1). Since the detection efficiency between different SOD1 mutants with antibody used in this study (polyclonal antibody raised against full-length SOD1 of human origin) may not necessarily be exactly equivalent, we could not completely exclude the possibility that expression levels of SOD1^G93A^ and SOD1^H46R^ are different. Nonetheless, considering comparable levels of both transcripts (Figure 1B and Table 1), it seems fair to conclude that their protein levels are also comparable. The results indicate that the expressions of the mutant SOD1 proteins are not affected by differences in mutations, genetic backgrounds, and/or genders in mice.

### Effects of different mutations on growth curves in *SOD1* transgenic mice with different genetic backgrounds

As all four congenic mouse lines generated in this study showed comparable levels of mutant transgene and protein expressions (Figure 1, Figure 2, and Table 1), it is assumed that these mutant *SOD1*-expressing ALS mouse models could be an appropriate means to analyze the effects of mutations, genetic backgrounds, and/or genders on gross phenotypes *in vivo*. In this study, we first focused on body weight, as it has been widely accepted that onset of disease in mutant-SOD1 transgenic ALS mouse models can be estimated by the reduction of body weight [24], [25].

**Figure 3:**
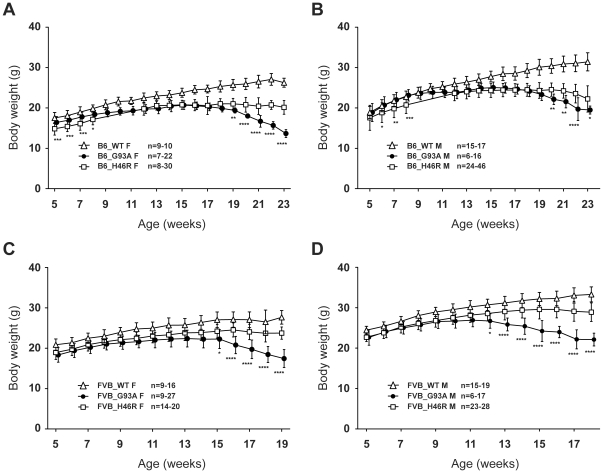
Growth curves for two different mutant *SOD1* transgenic mouse lines with different genetic backgrounds. (A) Growth curves for C57BL/6 (B6) congenic female mice [wild-type (B6_WT F; open triangle, n = 9–10), *SOD1^G93A^* (B6_G93A F; closed circle, n = 7–22), *SOD1^H46R^* (B6_H46R F; open square, n = 8–30)]. (B) Growth curves for B6 congenic male mice [wild-type (B6_WT M; open triangle, n = 15–17), *SOD1^G93A^* (B6_G93A M; closed circle, n = 6–16), *SOD1^H46R^* (B6_H46R M; open square, n = 24–46)]. (C) Growth curves for FVB/N (FVB) congenic female mice [wild-type (FVB_WT F; open triangle, n = 9–16), *SOD1^G93A^* (FVB_G93A F; closed circle, n = 9–27), *SOD1^H46R^* (FVB_H46R F; open square, n = 14–20)]. (D) Growth curves for FVB congenic male mice [wild-type (FVB_WT M; open triangle, n = 15–19), *SOD1^G93A^* (FVB_G93A M; closed circle, n = 6–17), *SOD1^H46R^* (FVB_H46R M; open square, n = 23–28)]. (A–D) Data are omitted from analysis when the numbers of live animals of the particular genotype at a particular age are <6. Values are mean ± SD. Statistical significances are evaluated by ANOVA with Bonferroni's *post hoc* test. In either gender or genetic background, degrees of body weight loss associated with disease progression in *SOD1^G93A^* mice are greater than those in *SOD1^H46R^* mice (*p<0.05, **p<0.01, ***p<0.001, ****p<0.0001). The values either for *SOD1^G93A^* or *SOD1^H46R^* mice, except for male B6 *SOD1^G93A^* mice earlier than 9 weeks of ages, are all significantly lower than those for age-matched WT animals (levels of significance are not shown).

During the experimental periods, both B6 and FVB congenic wild-type animals showed a constant increase in their body weight, whereas all the mutant SOD1 mouse lines started losing weight in the middle (Figure 3A–D). Indeed, both *SOD1^G93A^* and *SOD1^H46R^* mice with different genetic background exhibited progressive motor dysfunction and paralysis particularly on the phase of weight reduction (data not shown). The mean values of body weight for wild-type animals at each time point were significantly higher than those for mutant litters, except for those at earlier ages (5–9 weeks) of male B6_G93A (Figure 3B). The peak mean value of the body weight in each experimental group ranged from 11 to 18 weeks; 15 week in female B6_G93A and 15 week in female B6_H46R (Figure 3A), 15 week in male B6_G93A and 18 week in male B6_H46R (Figure 3B), 13 week in female FVB_G93A and 16 week in female FVB_H46R (Figure 3C), and 11 week in male FVB_G93A and 16 week in male FVB_H46R (Figure 3D). Notably, *SOD1^G93A^* mice showed more extensive reduction when compared to *SOD1^H46R^* animals irrespective of gender and genetic background (Figure 3A–D). Further, both female and male FVB_G93A exhibited an earlier body weight loss than FVB_H46R counterparts (Figure 3C and D). These results support the notion that the H46R mutation in *SOD1* results in a milder disease phenotype than does the G93A mutation in mouse [18], [22] and human [20]. On the other hand, visual inspection of animal behavior revealed that the onset of disease and the turning point of the growth curves were closely matched in B6 congenic mutant *SOD1* transgenic mice, as previously reported [24], [25]. However, it was obvious that the body weight were persistently increased at an early phase of disease progression, where animals clearly showed gait abnormalities, in FVB congenic mutant animals, particularly in FVB_H46R. Thus, the peak of the growth curve in mice on a FVB background may not define an earliest sign of disease.

### Effects of gender on survival in *SOD1^G93A^* and *SOD1^H46R^* transgenic mouse lines with different genetic backgrounds

**Figure 4:**
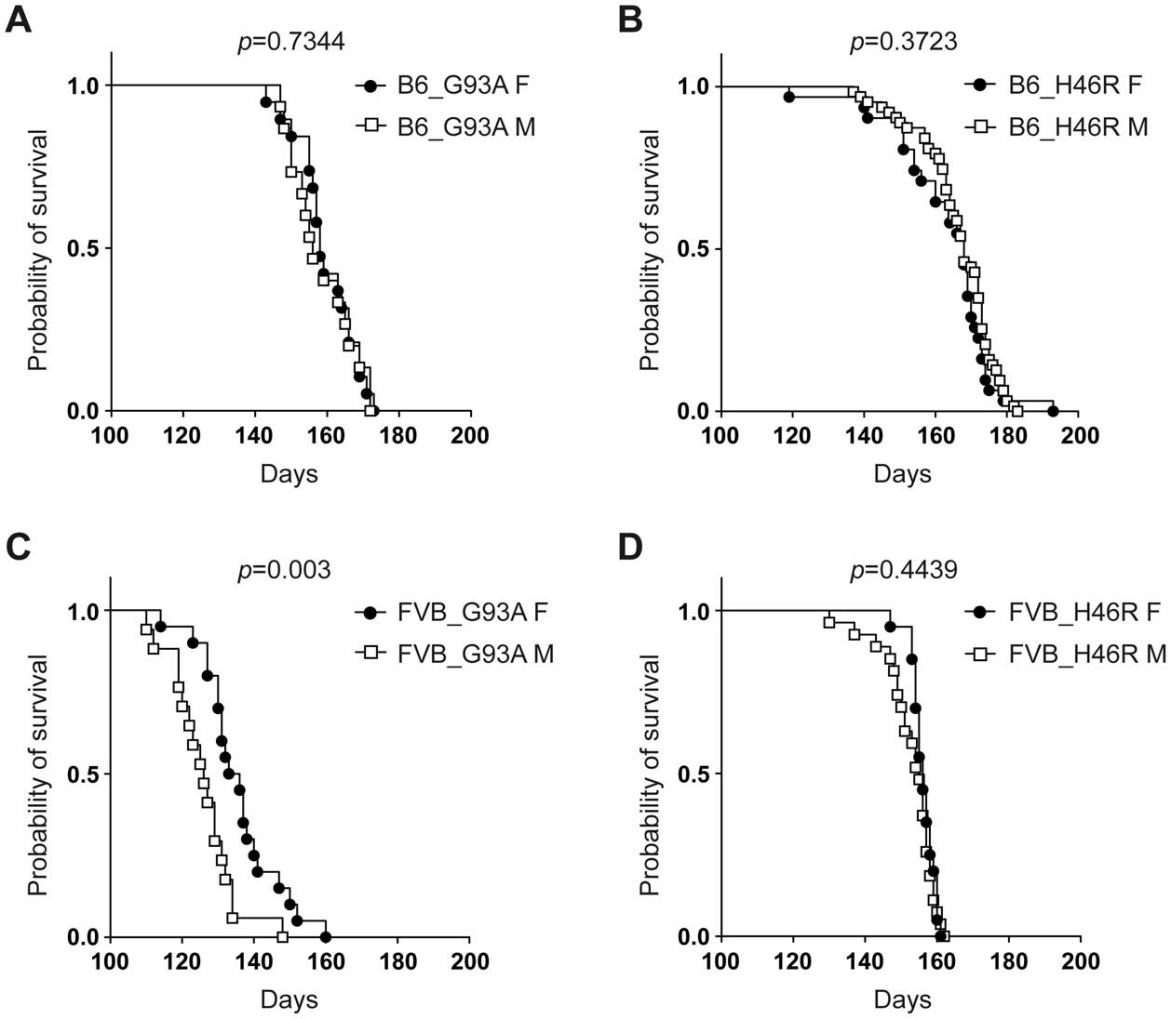
Effect of gender on survival in different mutant *SOD1* transgenic mouse lines. (A) Survival curves for C57BL/6 (B6) congenic *SOD1^G93A^* transgenic mice (B6_G93A) [female (F); closed circle: n = 19, male (M); open square: n = 15]. (B) Survival curves for B6 congenic *SOD1^H46R^* transgenic mice (B6_H46R) (F; closed circle: n = 31, M; open square: n = 63). (C) Survival curves for FVB/N (FVB) congenic *SOD1^G93A^* transgenic mice (FVB_G93A) (F; closed circle: n = 20, M; open square: n = 17). (D) Survival curves for FVB congenic *SOD1^H46R^* transgenic mice (FVB_H46R) (F; closed circle: n = 20, M; open square: n = 27). Kaplan-Meier analysis with Log-rank (Mantel-Cox) test reveals a significant gender difference in FVB_G93A (*p* = 0.003), but not in B6_G93A (*p* = 0.7344), B6_H46R (*p* = 0.3723), and FVB_H46R (*p* = 0.4439).

We next focused on lifespan (survival), which is thought to be one of the most important gross phenotype in ALS mouse models. The mean values of survival varied in mice with different *SOD1* mutations, genetic backgrounds, and/or genders (Table 2), consistent with previous reports [11], [16]. Kaplan-Meier survival analysis revealed that female FVB_G93A mice lived longer than male counterpart (Figure 4C). By contrast, no obvious gender effects on survival in other mouse lines, including B6_G93A (Figure 4A), B6_H46R (Figure 4B), and FVB_H46R mice (Figure 4D), were observed. These data suggest that gender differently affects survival in mice carrying the different *SOD1* mutation on a different genetic background.

**Table 2:** Lifespan of the mutant *SOD1* transgenic mouse lines used in this study.

Strain	Genotype	Gender	n	Lifespan (Survival)
				(days)*
B6	*SOD1^G93A^*	F	19	159.8±8.2
		M	15	158.6±8.7
B6	*SOD1^H46R^*	F	31	163.4±13.9
		M	63	166.6±10.6
FVB	*SOD1^G93A^*	F	20	135.8±10.7
		M	17	125.9±9.0
FVB	*SOD1^H46R^*	F	20	156.1±3.3
		M	27	152.9±7.3
FVB	*SOD1^G93A^*;*Als2* ^−/−^	F	13	133.2±9.9
		M	8	126.4±7.3
FVB	*SOD1^H46R^*;*Als2* ^−/−^	F	12	147.8±4.9
		M	10	142.5±12.3

* Values are mean ± SD.

### Effects of genetic background on survival in *SOD1^G93A^* and *SOD1^H46R^* transgenic mouse lines

**Figure 5:**
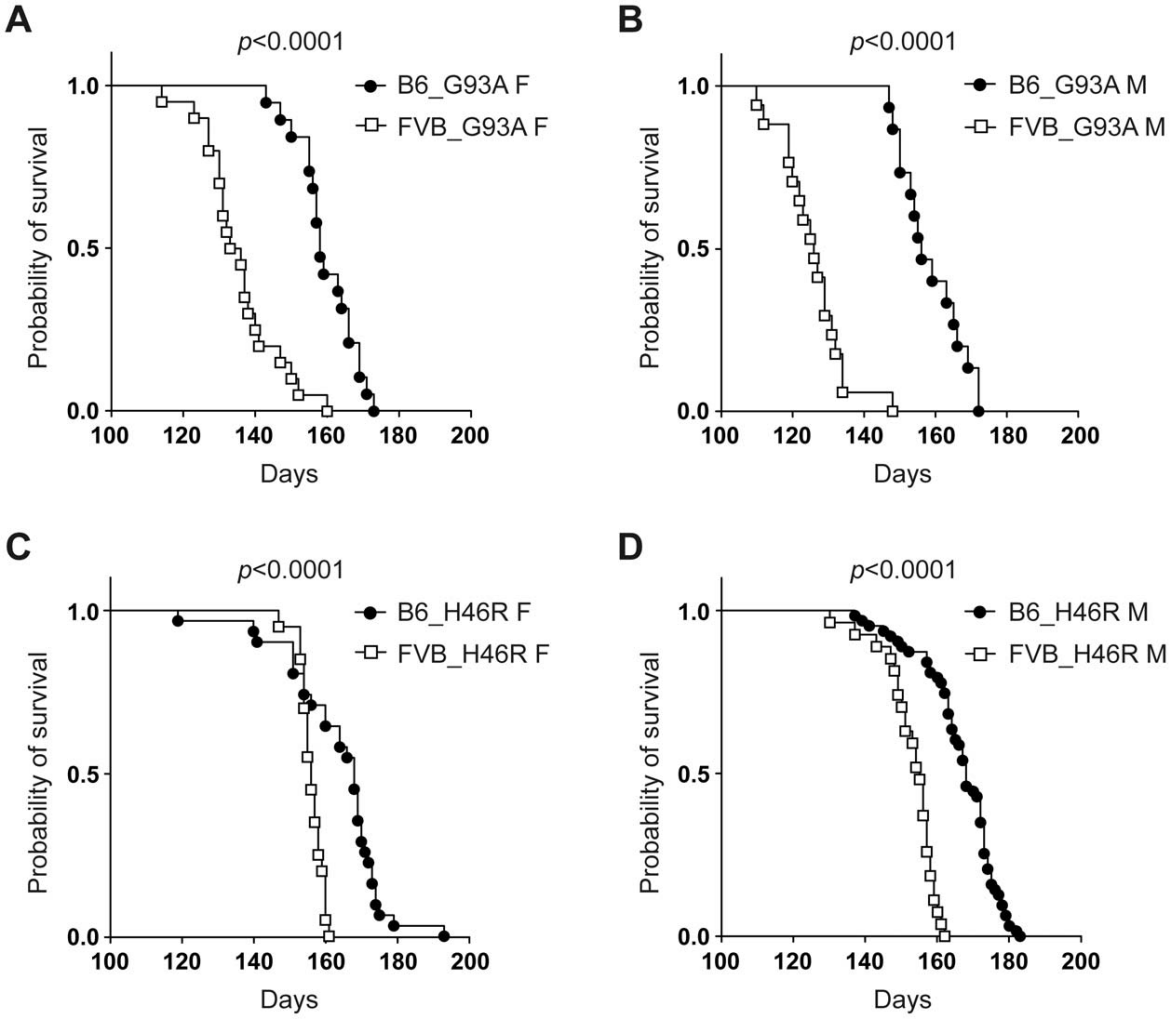
Effect of genetic background on survival in different mutant *SOD1* transgenic mouse lines. (A) Survival curves for C57BL/6 (B6) congenic *SOD1^G93A^* transgenic female mice (B6_G93A F) (closed circle: n = 19) and FVB/N (FVB) congenic *SOD1^G93A^* transgenic female mice (FVB_G93A F) (open square: n = 20). (B) Survival curves for B6 congenic *SOD1^G93A^* transgenic male mice (B6_G93A M) (closed circle: n = 15) and FVB congenic *SOD1^G93A^* transgenic male mice (FVB_G93A M) (open square: n = 17). (C) Survival curves for B6 congenic *SOD1^H46R^* transgenic female mice (B6_H46R F) (closed circle: n = 31) and FVB congenic *SOD1^H46R^* transgenic female mice (FVB_H46R F) (open square: n = 20). (D) Survival curves for B6 congenic *SOD1^H46R^* transgenic male mice (B6_H46R M) (closed circle: n = 63) and FVB congenic *SOD1^H46R^* transgenic male mice (FVB_H46R M) (open square: n = 27). Kaplan-Meier analysis with Log-rank (Mantel-Cox) test identifies significant differences in survival for mutant SOD1 transgenic lines between B6 and FVB backgrounds (*p*<0.0001).

We investigated whether genetic background affected on survival in our congenic mouse lines. Kaplan-Meier survival analysis revealed that all FVB congenic lines carrying mutant *SOD1* gene irrespective of their mutation and gender differences showed a significant shorter lifespan when compared to corresponding B6 lines (Figure 5A–D). Namely, compared to the H46R mutation (Figure 5C and D), devastating effects of the G93A mutation were more prominent in FVB congenic mice (Figure 5A and B). These results strongly indicate that difference in genetic background strongly affects lifespan in mutant *SOD1*-expressing ALS mouse models, and suggest that FVB congenic mice are more susceptible to mutant SOD1-mediated toxic insults than B6 lines.

### Effects of different types of the *SOD1* mutation on survival in transgenic mice with different genetic backgrounds

**Figure 6:**
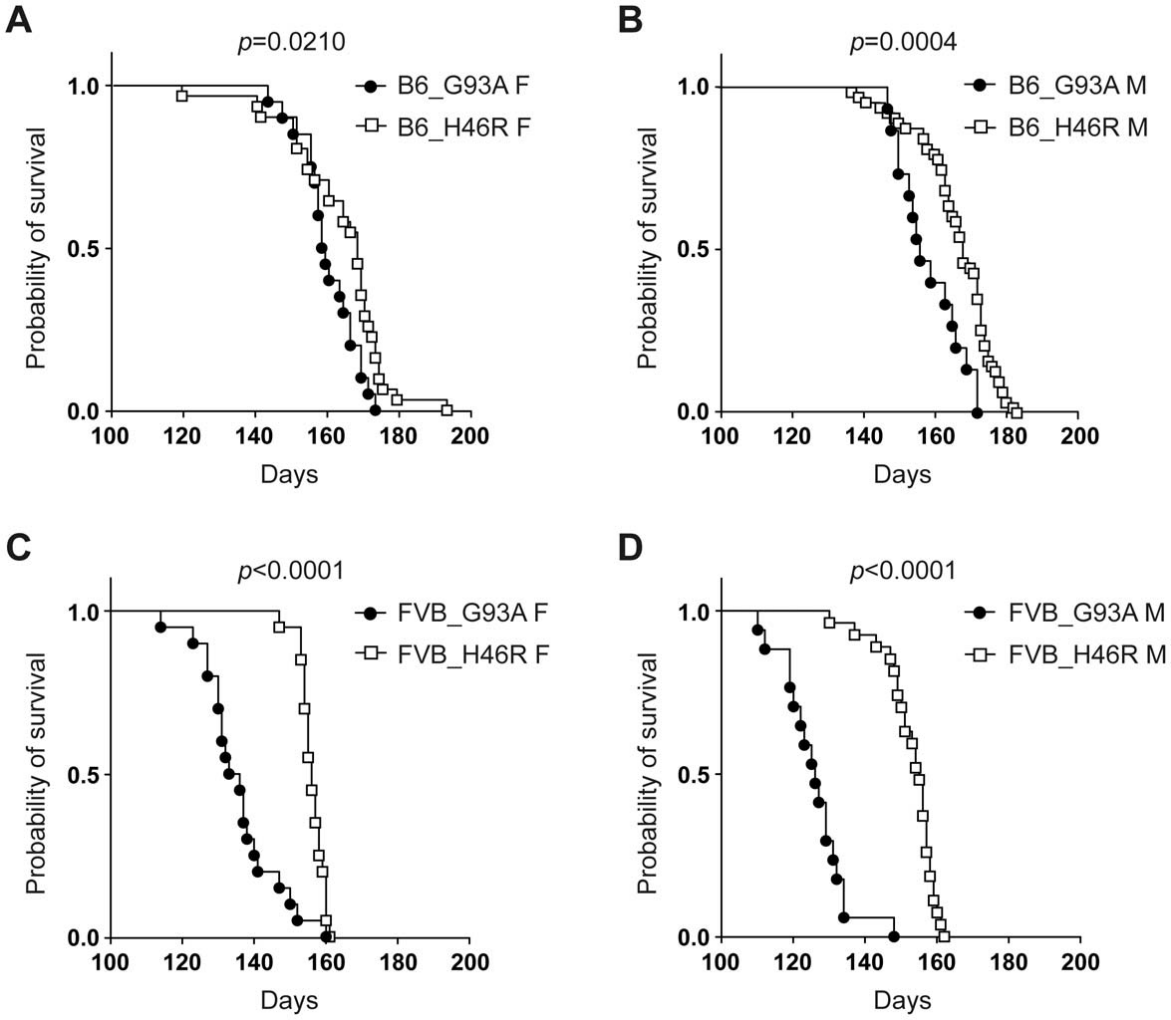
Effect of different types of the *SOD1* mutation on survival. (A) Survival curves for C57BL/6 (B6) congenic *SOD1^G93A^* transgenic female mice (B6_G93A F) (closed circle: n = 19) and B6 congenic *SOD1^H46R^* transgenic female mice (B6_H46R F) (open square: n = 31). (B) Survival curves for B6 congenic *SOD1^G93A^* transgenic male mice (B6_G93A M) (closed circle: n = 15) and B6 congenic *SOD1^H46R^* transgenic male mice (B6_H46R M) (open square: n = 63). (C) Survival curves for FVB/N (FVB) congenic *SOD1^G93A^* transgenic female mice (FVB_G93A F) (closed circle: n = 20) and FVB congenic *SOD1^H46R^* transgenic female mice (FVB_H46R F) (open square: n = 20). (D) Survival curves for FVB congenic *SOD1^G93A^* transgenic male mice (FVB_G93A M) (closed circle: n = 17) and FVB congenic *SOD1^H46R^* transgenic male mice (FVB_H46R M) (open square: n = 27). Kaplan-Meier analysis with Log-rank (Mantel-Cox) test identifies significant differences in survival between B6_G93A F and B6_H46R F (*p* = 0.0210), B6_G93A M and B6_H46R M (*p* = 0.0004), FVB_G93A F and FVB_H46R F (*p*<0.0001), and FVB_G93A M and FVB_H46R M (*p*<0.0001).

We next examined whether the different *SOD1* mutations affected survival in mice on the same genetic background and gender. Kaplan-Meier survival analysis revealed that the G93A mutation resulted in a shorter lifespan than did the H46R mutation in both B6 and FVB lines irrespective of gender (Figure 6A–D). It is noted that such toxic effects of the G93A mutation were more obvious in mice on a FVB background (Figure 6C and D). The data suggest that the G93A mutation in *SOD1* causes a severer disease phenotype than does the H46R mutation, and support the notion that FVB congenic mice are more susceptible to the *SOD1^G93A^*-mediated toxic insults than B6 lines.

### Effect of ALS2 loss on growth curves in FVB congenic *SOD1^G93A^* and *SOD1^H46R^* transgenic mouse lines

Loss of function mutation in the *ALS2* gene accounts for a number of juvenile recessive forms of ALS/MNDs [19], [26], [27]. Previously, we demonstrated that genetic ablation of *Als2* in *SOD1^H46R^*, but not *SOD1^G93A^*, mice on a B6 background aggravated the mutant SOD1-associated disease symptoms and led to the earlier death [18], suggesting distinctive susceptibilities to ALS2 loss in different mutant *SOD1*-expressing ALS mouse models. In this study, to clarify whether such functional interactions *in vivo* between ALS2 and mutant SOD1 depended on genetic background of mouse lines, we crossed FVB congenic *SOD1^G93A^* and *SOD1^H46R^* mice to FVB congenic *Als2*-null mice [28], generating FVB congenic ALS2-deficient *SOD1^G93A^* (*Als2* ^−/−^;*SOD1^G93A^*) and *SOD1^H46R^* (*Als2* ^−/−^;*SOD1^H46R^*) mice (FVB_G93A_Als2−/− and FVB_H46R_Als2−/−), respectively, and analyzed their body weight and lifespan.

**Figure 7:**
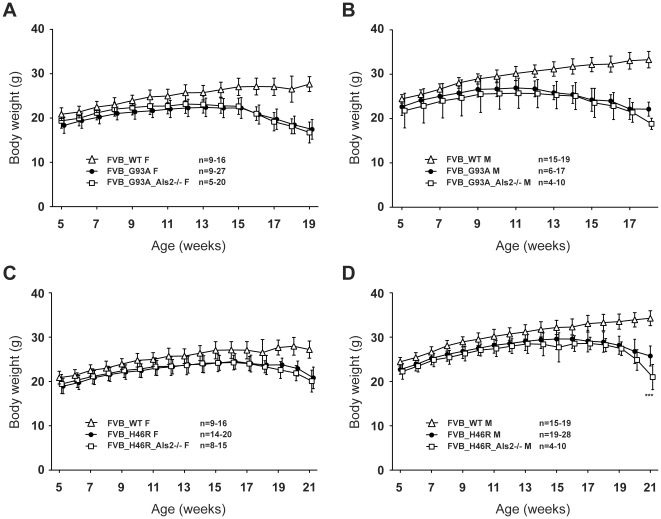
Growth curves for ALS2 deficient *SOD1* transgenic mice on a FVB background. (A) Growth curves for FVB/N (FVB) congenic female mice [wild-type (FVB_WT F; open triangle, n = 9–16), *SOD1^G93A^* (FVB_G93A F; closed circle, n = 9–27), *SOD1^G93A^*;*Als2* ^−/−^ (FVB_G93A_Als2−/− F; open square, n = 5–20)]. (B) Growth curves for FVB congenic male mice [wild-type (FVB_WT M; open triangle, n = 15–19), *SOD1^G93A^* (FVB_G93A M; closed circle, n = 6–17), *SOD1^G93A^*;*Als2* ^−/−^ (FVB_G93A_Als2−/− M; open square, n = 4–10)]. (C) Growth curves for FVB congenic female mice [wild-type (FVB_WT F; open triangle, n = 9–16), *SOD1^H46R^* (FVB_H46R F; closed circle, n = 14–20), *SOD1^H46R^*;*Als2* ^−/−^ (FVB_H46R_Als2−/− F; open square, n = 8–15)]. (D) Growth curves for FVB congenic male mice [wild-type (FVB_WT M; open triangle, n = 15–19), *SOD1^H46R^* (FVB_H46R M; closed circle, n = 19–28), *SOD1^H46R^*;*Als2* ^−/−^ (FVB_H46R_Als2−/− M; open square, n = 4–10)]. (A–D) Data are omitted from analysis when the numbers of live animals of the particular genotype at a particular age are <4. Values are mean ± SD. Statistical significances are evaluated by ANOVA with Bonferroni's *post hoc* test. Degree of body weight loss associated with disease progression in male FVB *SOD1^H46R^* mice is greater than that in male FVB *SOD1^H46R^*;*Als2* ^−/−^SOD1^H46R^ mice (***p<0.001). The values for all mutant SOD1 transgenic mice with or without ALS2 are significantly lower than those for age-matched WT animals (levels of significance are not shown).

During the experimental periods, the mean values of body weight for FVB congenic wild-type animals at each time point were significantly higher than those for all mutant litters (Figure 7A–D), consistent with the previous findings observed in B6 congenic lines [18]. There were no significant differences in body weight between both mutant *SOD1* mice and their ALS2-deficient counterparts (Figure 7A–D), except that male FVB_H46R_Als2−/− showed a lower value than male FVB_H46R at 21 weeks of age (Figure 7D). The results suggest a limited role of ALS2 in growth curve in FVB congenic mutant *SOD1*-expressing mice.

### Effect of ALS2 loss on survival in FVB congenic *SOD1^G93A^* and *SOD1^H46R^* transgenic mouse lines

**Figure 8:**
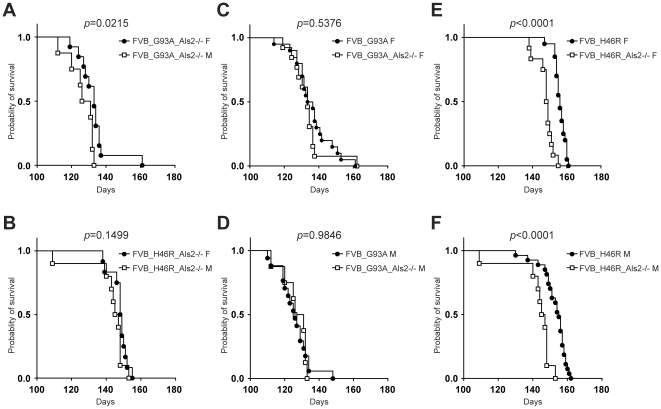
Effect of ALS2 loss on survival in FVB congenic *SOD1* transgenic mice. (A) Survival curves for FVB/N (FVB) congenic *SOD1^G93A^*;*Als2* ^−/−^ mice (FVB_G93A_Als2−/−) [female (F); closed circle: n = 13, male (M); open square: n = 8]. (B) Survival curves for FVB congenic *SOD1^H46R^*;*Als2* ^−/−^ mice (FVB_H46R_Als2−/−) (F; closed circle: n = 12, male (M); open square: n = 10). (C) Survival curves for FVB/N (FVB) congenic *SOD1^G93A^* transgenic female mice (FVB_G93A F) (closed circle: n = 20) and FVB congenic *SOD1^G93A^*;*Als2* ^−/−^ female mice (FVB_G93A_Als2−/− F) (open square: n = 12). (D) Survival curves for FVB congenic *SOD1^G93A^* transgenic male mice (FVB_G93A M) (closed circle: n = 17) and FVB congenic *SOD1^G93A^*;*Als2* ^−/−^ male mice (FVB_G93A_Als2−/− M) (open square: n = 8). (E) Survival curves for FVB congenic *SOD1^H46R^* transgenic female mice (FVB_H46R F) (closed circle: n = 20) and FVB congenic *SOD1^H46R^*;*Als2* ^−/−^ female mice (FVB_H46R_Als2−/− F) (open square: n = 12). (F) Survival curves for FVB congenic *SOD1^H46R^* transgenic male mice (FVB_H46R M) (closed circle: n = 27) and FVB congenic *SOD1^H46R^*;*Als2* ^−/−^ male mice (FVB_H46R_Als2−/− M) (open square: n = 10). Kaplan-Meier analysis with Log-rank (Mantel-Cox) test reveals a significant gender difference in FVB_G93A_Als2−/− (*p* = 0.0215), but not in FVB_H46R_Als2−/− (*p* = 0.1499) (A, B). While no statistical differences between FVB_G93A and FVB_G93A_Als2−/− in both genders are observed (C, D), both female and male ALS2 deficient FVB_H46R mice exhibit significant shorter lifespans than corresponding FVB_H46R mice (*p*<0.0001) (E, F).

To further investigate the effect of ALS2 loss on survival in FVB congenic *SOD1^G93A^* and *SOD1^H46R^* transgenic mouse lines, we analyzed lifespan by Kaplan-Meier analysis. Consistent with the observation in FVB_G93A and FVB_H46R mice (Figure 4C and D), there was a small but significant gender difference in FVB_G93A_Als2−/− (*p* = 0.0215) (Figure 8A), while no differences in FVB_H46R_Als2−/− mice were observed (Figure 8B). Importantly, as in the case for B6 lines [18], FVB_H46R_Als2−/− mice died earlier than FVB_H46R mice in both gender (Figure 8E and F), while no differences in lifespan between FVB_G93A_Als2−/− and FVB_G93A mice were observed (Figure 8C and D). These results combined with previous findings [18] suggest a distinctive susceptibility to ALS2 loss in different mutant *SOD1*-expressing mice irrespective of their genetic background, and indicate that the pathogenic pathways associated with ALS2 loss and SOD1^H46R^, but not SOD1^G93A^, -mediated neuronal toxicity might act synergistically *in vivo*.

## Discussion

In the present study, we generated four congenic ALS mouse model lines; *SOD1^G93A^* and *SOD1^H46R^* mice on two different genetic backgrounds; B6 and FVB, and showed that the expression levels of the mutant SOD1 proteins among these different lines were comparable. This allows us to conduct a comparative analysis of growth curve and survival using these mice, demonstrating some concrete evidence indicating that two different SOD1 mutations exerts a distinct harmful effect on gross phenotypes in mice.

Multiple epidemiological surveys have indicated that gender affects the incidence, age at onset, and disease duration in sALS patients [29], [30]. However, the male-to-female ratio in the SOD1-linked fALS patients is 1∶1 [3], rather suggesting the absence of gender effects in human ALS patients with the *SOD1* mutations. Nonetheless, it has been reported that female mice lived longer than male in several different congenic *SOD1^G93A^* lines on a SJL/J (SJL), C3H/HeJ (C3H), BALB/cByJ (BALB), and FVB backgrounds, while neither B6 nor DBA/2J (DBA) congenic mice showed any gender effects on lifespan [15], [16]. Consistently, we here showed that female FVB, but not B6, congenic *SOD1^G93A^* mice lived longer than corresponding male counterpart, confirming that the G93A mutation does exert a genetic background-dependent gender-specific harmful effect in mice. Interestingly, unlike *SOD1^G93A^* mice, both B6 and FVB congenic mice expressing *SOD1^H46R^* showed no observable gender effect on survival. Together, not only genetic background but also difference in the *SOD1* mutations may affect the gender-associated phenotypic modification, at least, in mice.

In addition to gender effects as above, it is widely appreciated that genetic background affects many phenotypes including lifespan in mutant SOD1-expressing ALS mouse models [15],[16],[31],[32]. Likewise, *Als2*-null mice, another type of ALS/MND model, on a FVB but not B6 background shows shorter lifespan than do wild-type litters [28]. Consistently, we here revealed that both FVB congenic *SOD1^G93A^* and *SOD1^H46R^* lines exhibited a significant shorter lifespan when compared to B6 counterparts. It is noted that the mutant SOD1-mediating devastating effects on life span appear more prominent in *SOD1^G93A^* than *SOD1^H46R^* mice, particularly those on a FVB background. FVB mice are known to be more vulnerable to glutamate receptor-mediated excitotoxic cell death [33], [34], as well as to mitochondrial toxin-mediated metabolic cell death [35], when compared to B6 mice. These findings suggest that FVB congenic mice may have certain properties involving in the preferential vulnerability to cellular-toxicities including mutant SOD1-mediated insults. Conversely, it is equally likely that B6 mouse carries the gene conferring the resistance to these insults.

One of the important findings obtained from this study was that the different SOD1 mutations showed distinct adverse effects on gross phenotypes in ALS/MND mouse models. Comparative analysis of growth curves and lifespans revealed that the G93A mutation in *SOD1* caused a severer disease phenotype than did the H46R mutation, where *SOD1^G93A^* mice showed more extensive body weight loss and earlier death. These trends were more striking in FVB congenic mice. Remarkably, lack of *Als2*, a murine homolog for the recessive juvenile ALS causative gene [19], in FVB congenic *SOD1^H46R^*, but not *SOD1^G93A^*, mice resulted in an earlier death. We have previously demonstrated similar results using B6 congenic *Als2*-deficient *SOD1^H46R^* but not *SOD1^G93A^* mice [18], indicating a genetic background-independent but mutation-dependent phenotypic modification. Although the possibility that phenotypic variances observed are due to locus-specific effects of each transgene and/or adjacent genes in a particular genomic region should not be excluded, these findings lend credence to the notion that SOD1^G93A^- and SOD1^H46R^-mediated toxicity and their associated pathogenic pathways are not identical. Recent findings that loss of *Gfap*, a gene encoding glial fibrillary acidic protein (GFAP), in B6 congenic *SOD1^H46R^*, but not *SOD1^G93A^*, mice slightly accelerates the disease progression [22], also support this idea.

Provided that the SOD1^G93A^ and SOD1^H46R^ mutants exert the distinctive harmful effects on symptoms associated with ALS/MNDs, how does different mutation in the same gene result in the distinctive phenotypic modification? It has been reported that although age at onset among families carrying different *SOD1* mutations with high penetrance is less variable with the mean value ranging from 45 to 50 years [3], the mean disease duration after onset varies depending on mutation type, with a range of 0.9 to 18.7 years among *SOD1*-linked families [3]. Indeed, the disease durations after onset in patients with the G93A and H46R mutations are considerably different (G93A vs H46R; 2.2±1.5 vs 17.0±11.0 years) [3], [21]. It is noted that our congenic ALS mouse models partly recapitulate such differences. Currently, although exact molecular basis for the mutation-dependent effect remains unclear, it is appreciated that the SOD1-mediated dismutase enzymatic activity is not a major determinant for the phenotypic modification, since there is no correlation between disease severities and the SOD1 dismutase activities [36], [37]. Rather, the differences in the propensity for the aggregate formation among the different mutant SOD1 proteins might be related [7], [38]. In cultured cells, SOD1^H46R^ mutant forms fewer insoluble aggregates and inclusions when compared with SOD1^G93A^ [38]. Further, unlike in *SOD1^G93A^* mice [39], no obvious SOD1-positive inclusions are detected in the spinal cord of *SOD1^H46R^* mice [18]. These results indicate that SOD1^H46R^ is less prone to form aggregates than SOD1^G93A^. Additionally, large vacuolar structures originated from distended mitochondria are evident in *SOD1^G93A^* [12], while such pathological features are barely observed in *SOD1^H46R^* mice. Instead, a widespread axonal pathology and degeneration considerably precede motor neuron loss in the spinal cord of *SOD1^H46R^* mice [18], [23]. Thus, it is conceivable that molecular basis for the pathogenesis in each mutant SOD1-expressing mouse model may not be the same.

It is generally thought that symptomatic heterogeneity observed in patients with ALS/MNDs may reflect varied etiology and/or results from a complex interplay between other genetic and environmental factors [8], [9], [40]. Some epidemiological studies suggest that traumatic injury and exercise are risk factors for the development of ALS [41], [42], but these results were not widely confirmed [43]. Animal studies using a *SOD1^G93A^* ALS model have demonstrated that extensive endurance exercise hastens a decrease in motor performance and death following onset of disease in male mice [44], while moderate exercise seems to be beneficial [45]. Further, it has recently been shown that environmental enrichment significantly improves motor performance in *SOD1^G93A^* mice in a gender-specific manner [46]. However, it is still unclear as to whether and how such environmental factors affect disease symptoms. Future studies using different congenic ALS mouse models are warranted to clarify not only genetic but also such environmental factors associated with ALS/MNDs.

Preclinical animal studies are prerequisite for the development of therapeutic agents for the treatment of ALS/MNDs. Thus far, a large number of successful therapeutic interventions in preclinical animal studies have failed to translate into human applications [40]. To solve these issues, standard operation procedures (SOPs) for preclinical animal studies for ALS/MNDs has recently been proposed [47], in which in addition to the minimum experimental requirements for any proof of concept or preclinical study, the use of not only *SOD1^G93A^* model but also other potential ALS models such as transgenic mice expressing either mutant dynactin, TAR DNA-binding protein (TDP43), or fused in sarcoma (FUS) mutants are recommended. However, the trend is still largely biased toward the use of single mutant SOD1 ALS mouse model; i.e., *SOD1^G93A^* [47]. We here propose that mice expressing SOD1 mutants other than *SOD1^G93A^* should also be included within this list. It seems logical that convincing evidences could be obtained by replicated demonstrations of efficacy and effectiveness of interventions with different ALS models in preclinical animal studies.

In conclusion, the findings presented in this study strongly support the notion that the different mutant SOD1 proteins cause motor neuron dysfunction and death by a similar but not identical mechanism, suggesting the presence of different genetic modifiers of disease expression, which are associated with a combination with particular SOD1 mutation, genetic background, and/or gender. Thus, congenic ALS mouse models with different SOD1 mutations generated in this study should provide a useful means not only for the identification of modifier genes as well as environmental factors associated with ALS/MNDs, but also for preclinical animal studies in ALS/MNDs.

## Materials and Methods

### Ethics statement

All animal experimental procedures were carried out in accord with the Fundamental Guidelines for Proper Conduct of Animal Experiment and Related Activities in Academic Research Institutions under the jurisdiction of the Ministry of Education, Culture, Sports, Science and Technology, Japan, and reviewed and approved by The Institutional Animal Care and Use Committee at Tokai University.

### Animals

We generated C57BL/6N (B6) congenic *SOD1^H46R^* mice by crossing original *SOD1^H46R^*-tg males (C57BL/6×DBA/2) [23] to B6 females for more than 16 generations (>N16). Then, B6 congenic *SOD1^H46R^*-tg males were backcrossed to FVB/NJcl (FVB) females for more than 10 generations (>N10), generating FVB congenic *SOD1^H46R^* lines. Congenic *SOD1^G93A^* mice on two different genetic backgrounds were also generated by crossing B6SJL-TgN(SOD1-G93A)1Gur males (C57BL/6J×SJL) [10] derived from Jackson Laboratories to either B6 or FVB females for more than 10 generations (>N10). In addition, we newly generated two FVB congenic lines of double mutants; *Als2* ^−/−^;*SOD1^H46R^* and *Als2* ^−/−^;*SOD1^G93A^*. FVB congenic lines of *Als2* ^+/−^ mice generated by crossing F2 *Als2* ^+/−^ mice (129P2×B6) [48] to FVB mice for more than 10 generations (>N10) [28] were utilized to produce FVB congenic *Als2* ^+/−^;*SOD1^H46R^* and *Als2* ^+/−^;*SOD1^G93A^* mice. We crossed FVB congenic *SOD1^H46R^* or *SOD1^G93A^* males to FVB congenic *Als2* ^+/−^ female mice, generating mice with nine different genotypes; *Als2* ^+/+^ (wild-type), *Als2* ^+/−^, *Als2* ^−/−^, *Als2* ^+/+^;*SOD1^H46R^*, *Als2* ^+/−^;*SOD1^H46R^*, *Als2* ^−/−^;*SOD1^H46R^*, *Als2* ^+/+^;*SOD1^G93A^*, *Als2* ^+/−^;*SOD1^G93A^*, and *Als2* ^−/−^;*SOD1^G93A^*, by crossing male *Als2* ^+/−^;*SOD1^H46R^* or *Als2* ^+/−^;*SOD1^G93A^* to female *Als2* ^+/−^ mice. Among these animals, wild-type, *Als2* ^+/+^;*SOD1^H46R^*, *Als2* ^−/−^;*SOD1^H46R^*, *Als2* ^+/+^;*SOD1^G93A^*, and *Als2* ^−/−^;*SOD1^G93A^* mice were used in this study. Mice were genotyped by PCR using genomic DNA from tail tissues as described [18], [48]. Mice were housed at 22–23°C with a 12 hr light/dark cycle. Food and water were fed *ad libitum*. Body weight of each animal was weekly monitored. Their lifespan (endpoint) was determined by the observation that mice were unable to move by themselves.

### Analysis of the copy number of the *SOD1* transgene

The copy numbers of the human *SOD1* transgene in the mouse genome was estimated using real time quantitative PCR by determining the difference in threshold cycle (ΔCT) between the human *SOD1* transgene and a reference mouse gene (*Sod1*). Primers used in this study were as follows: human *SOD1*; forward (F): 5′-TGCCAGCAGAGTACACAAG-3′, reverse (R): 5′-ATCAAAGCCCAGTTTTGTGG-3′, mouse *Sod1*; F: 5′-GATTGGGTTTGACCCATTTG-3′, R: 5′-GCTCAACAATGCAGCAAGTC-3′. The real time PCR was performed in a 20 µl reaction mixture containing genomic DNA (10 ng), primers mix (final primer concentration of 500 nM each), and SYBR Green PCR Master Mix (QuantiFast SYBR Green PCR Kit; Qiagen) in a MicroAmp 96 well plate (Applied Biosystems) using 7500 Fast Real-Time PCR System (Applied Biosystems). The thermal conditions were the initial denaturation at 95°C for 5 min followed by 40 cycles of 95°C for 10 sec and 60°C for 30 sec.

### Preparation protein and total RNA samples

Spinal cord tissues were weighed and homogenized in 2 weight-volume (mg/µl) of phosphate buffer saline (PBS). A fraction (25 µl) of the homogenates was subjected to RNA extraction, and the remaining of them was used for protein extraction as described [22]. In brief, total RNA was extracted from tissue homogenates using Sepazole RNA I super G (Nacalai Tesque), and purified by SV Total RNA Isolation System (Promega) according to the manufacturer's instructions. Protein samples were obtained from the remaining tissue homogenates by lysing with buffer A [25 mM Tris-HCl (pH 7.5), 50 mM NaCl, 1% (w/v) Triton X-100 (TX), Complete Protease Inhibitor Cocktail (Roche)], followed by centrifugation at 23,000×g for 20 min at 4°C. The resultant supernatant was collected as a TX-soluble protein fraction. Protein concentration of each fraction was determined by the Micro BCA or Pierce 660 nm Protein Assay system (Thermo Scientific).

### Quantitative reverse transcriptase-PCR

The quantitative reverse transcriptase (qRT)-PCR was performed on a 0.5 ng of total RNA using QuantiFast SYBR Green RT-PCR (Qiagen) with specific primers (0.6 µM each) as follows; human *SOD1*; F: 5′-AGGGCATCATCAATTTCGAG-3′, R: 5′-ACATTGCCCAAGTCTCCAAC-3′, mouse *Actb* (β-actin); F: 5′-AGCCATGTACGTAGCCATCC-3′, R: 5′-TCTCAGCTGTGGTGGTGAAG-3′. The thermal cycling conditions consisted of a 30 min of reverse transcription step at 50°C, 10 min of initial denaturation at 95°C, followed by 40 cycles of amplification steps of 95°C for 15 sec and 60°C for 30 sec. The levels of the *SOD1* transcripts were analyzed by determining the difference in ΔCT between the human *SOD1* and the mouse *Actb* transcripts in each sample.

### Antibodies and western blot analysis

Primary antibodies used for western blot analysis included rabbit polyclonal anti-SOD1 (1∶50,000, Santa Cruz, FL-154) and anti-β-actin (1∶1000, Sigma, A-5060) antibodies. Secondary antibody was horseradish peroxidase (HRP)-conjugated donkey anti-rabbit IgG (1∶5000, Amersham Bioscience). Equal amount of proteins (1–2 µg) were electrophoretically separated by a SDS-polyacrylamide gel (SuperSep Ace, 5–20% WAKO) and transferred onto a polyvinylidine difluride membrane (Bio-Rad). The membranes were blocked with Blocking One (Nacalai Tesque) for 2 hr at room temperature, and incubated with the appropriate primary antibody in TBST [20 mM Tris-HCl (pH 7.5), 150 mM NaCl, 0.1% (w/v) Tween 20] containing 5% Blocking One (Nacalai Tesque). After washing with TBST, membranes were incubated with HRP-conjugated secondary antibody at room temperature for 2 hr, followed by a repeated wash with TBST. Signals were visualized by Immobilon Western (Millipore) and X-ray film (Amersham Bioscience), and were quantified by analyzing the digitally captured images using CS Analyzer ver3 (ATTO).

### Statistical analysis

Statistical analyses were conducted using PRISM5 (GraphPad). Statistical significance was evaluated by ANOVA followed by appropriate *post hoc* tests for multiple comparisons between groups. Survival data were compared using Kaplan-Meier survival analysis with Log-rank (Mantel-Cox) test. A *p*-value<0.05 was considered as reaching statistical significance.
